# Adherence to the Mediterranean Diet, Sodium and Potassium Intake in People at a High Risk of Dementia

**DOI:** 10.3390/nu16101419

**Published:** 2024-05-08

**Authors:** Joana Rodrigues, Mariana Costa, Daniela de Sousa, Ana Rute Costa, Nuno Lunet, Vítor Tedim Cruz, Patrícia Padrão

**Affiliations:** 1 Faculdade de Ciências da Nutrição e Alimentação, Universidade do Porto, 4150-180 Porto, Portugal; up201809466@edu.fcna.up.pt (J.R.); up201804513@up.pt (M.C.); 2 EPIUnit—Instituto de Saúde Pública, Universidade do Porto, 4050-600 Porto, Portugal; daniela.sousa@ispup.up.pt (D.d.S.); arcosta@ispup.up.pt (A.R.C.); nlunet@med.up.pt (N.L.); vtedimcruz@gmail.com (V.T.C.); 3 Laboratório para a Investigação Integrativa e Translacional em Saúde Populacional (ITR), 4050-600 Porto, Portugal; 4 Departamento de Saúde Pública e Ciências Forenses e Educação Médica, Faculdade de Medicina, Universidade do Porto, 4200-319 Porto, Portugal; 5 Serviço de Neurologia, Unidade Local de Saúde de Matosinhos, 4464-513 Senhora da Hora, Portugal

**Keywords:** Mediterranean diet, salt, sodium, potassium, dementia

## Abstract

Adequate sodium and potassium intake, along with adherence to the Mediterranean diet (MedDiet), are key factors for preventing hypertension and cerebrovascular diseases. However, data on the consumption of these nutrients within the MedDiet are scarce. This cross-sectional study aims to assess the association between MedDiet adherence and sodium/potassium intake in the MIND-Matosinhos randomized controlled trial, targeting Portuguese adults at a high risk of dementia. Good adherence to the MedDiet was defined using the Portuguese Mediterranean Diet Adherence Screener questionnaire (≥10 points), and both sodium/potassium intakes were estimated from 24-hour urine collections. The association between MedDiet adherence and these nutrients’ intake (dichotomized by the median) was quantified by calculating odds ratios (OR) and respective 95% confidence intervals (95% CI) using a logistic regression. A total of 169 individuals (60.9% female; median age: 70 years; range: 36–85 years) were included. Good adherence to the MedDiet was observed among 18.3% of the sample. After adjusting for sex, age, education and using antihypertensive drugs, good MedDiet adherence was associated with higher sodium (OR = 3.11; 95% CI: 1.27–7.65) and potassium intake (OR = 9.74; 95% CI: 3.14–30.26). Increased adherence to the MedDiet may contribute to a higher potassium intake but seems to have limited effects on the adequacy of sodium levels.

## 1. Introduction

Excessive sodium intake and insufficient potassium intake are associated with the development of a variety of comorbidities, including hypertension, a major risk factor for cardiovascular [1] and cerebrovascular diseases [2], which, combined with vascular risk factors, can lead to a wide spectrum of cognitive disorders [3]. This is a topic of great relevance, as the prevalence of dementia has been increasing worldwide [4], and Portugal is expected to reach 350 thousand patients by 2050 [4].

The World Health Organization (WHO) suggests a maximum daily sodium intake of two grams (equivalent to five grams of salt for adults [5]) and a daily potassium consumption of at least 3.51 g to reduce blood pressure in adults [6]. Hence, a sodium-to-potassium ratio (Na/K ratio) equal to or below 1.0 is considered beneficial for cardiovascular health [7]. However, the Portuguese population has a mean daily intake of more than double the recommended amount for sodium [8] and a mean daily potassium intake lower than the recommended [9].

Data from a national study, conducted in 2015, also showed a prevalence of hyper-tension of 36.0% and 71.3% among Portuguese adults and older people, respectively [10], which reinforces the importance of studying its determinants, particularly among people at a high risk of dementia given that the deleterious association between hypertension and cognitive decline has been suggested in the literature [11,12]. This association is complex, and although cerebral small vessel disease and alterations in the autoregulatory capacity of the brain are pointed out as the main mechanisms involved in the relation between high blood pressure and cognitive decline, not all the potential mechanisms are completely clarified [13].

The traditional Mediterranean diet (MedDiet) is described as a high intake of plant foods (such as fruits, vegetables, pulses, nuts, bread and seeds); minimally processed, seasonally fresh, and locally grown foods; fresh fruit as a typical dessert, with sweets containing sugars or honey a few times per week; a high intake of extra-virgin olive oil used as the principal source of fat; a moderate intake of dairy products (mostly as cheese and yogurt); a moderate intake of eggs; fish and poultry consumed in low to moderate amounts; red meat consumed in low amounts and wine in moderation, consumed with meals [14].

Due to its plant-based nature and preference for minimally processed foods, it is expected that sodium and potassium intake may be adequate for those following a MedDiet. Good adherence to this dietary pattern and an adequate intake of these nutrients have been widely described as protective factors against hypertension, as well as cerebrovascular [15,16,17] and cardiovascular diseases [18,19,20,21].

Although the health benefits of good adherence to the MedDiet are well described in the literature, data regarding its association with sodium and potassium intake are still scarce and inconsistent. On the one hand, the MedDiet implies a high intake of food groups that naturally contain low amounts of sodium and high amounts of potassium, such as vegetables, fruits and pulses. On the other hand, this dietary pattern may be a source of hidden sodium through added salt during cooking or at the table and by including non-negligible quantities of traditionally processed products that are usually rich in salt [22]. Also, most food preservatives used by the industry today have a high sodium content and are major causes of an increased dietary intake of this nutrient [1].

Despite the increase in studies addressing the benefits of the MedDiet and the relevance of increased potassium and reduced sodium levels observed in recent years, the epidemiological evidence in favor of a direct association between the degree of adherence to the MedDiet and low habitual salt intake is not unequivocal [23]. Therefore, this study aims to quantify the association between the MedDiet and sodium and potassium intake among Portuguese adults at a high risk of dementia. To the best of our knowledge, this is the first study to evaluate this association in people at a high risk of dementia, which is expected to provide insights into the role of vascular health in cognitive function.

## 2. Materials and Methods

### 2.1. Study’s Design and Participants

This cross-sectional study used baseline data from the MIND-Matosinhos project (registration number: NCT05383443), a randomized controlled trial conducted in Matosinhos, Portugal, and designed to evaluate the effectiveness of a community-based program to prevent cognitive decline. Participants met the following criteria: (1) age between 18 and 85 years; (2) score on the Montreal Cognitive Assessment (MoCA) equal to or higher than the validated cut-off points defined as two standard deviations (SD) below the mean for the corresponding age and education in the Portuguese population [24]; (3) a high risk of developing dementia over the next 20 years based on the Cardiovascular Risk Factors, Aging and Dementia (CAIDE) [25] risk score (score ≥6 points) and (4) at least four years of schooling. The exclusion criteria included the existence of disorders or conditions in which exercise is contraindicated, lack of autonomy in performing daily activities and a confirmed diagnosis of dementia or major disability.

The MIND-Matosinhos project was conducted according to the guidelines established by the Declaration of Helsinki. The study protocol was approved by the Ethics Committee of the Matosinhos Local Health Unit (Ref. 63/CES/JAS and 71/CES/JAS). Approval was also obtained from the Data Protection Officer of the Institute of Public Health of the University of Porto. All study participants signed a duplicate informed consent form.

Participants at a high risk of dementia from the MIND-Matosinhos, evaluated at baseline between 2020 and 2023 (n = 207), were eligible for the present study. Individuals with missing data on adherence to the MedDiet (n = 2), those who did not perform the 24-hour urine collection (n = 25), those whose urine samples were considered incomplete (n = 10) or subjects whose sodium excretion was below the laboratory test’s detection limit (n = 1) were excluded. Therefore, a total of 169 participants providing complete 24-hour urine samples and adherence to the MedDiet data were included in this analysis.

### 2.2. Data Collection

Sociodemographic, lifestyle and health-related data, as well as physical activity and adherence to the MedDiet, were obtained through structured questionnaires conducted by trained interviewers.

#### 2.2.1. Sociodemographic Data

Sociodemographic data included sex, age, educational level, marital status, household income and professional status. Age was categorized as <65, 65 to 74 and ≥75 years. Educational level was determined by the number of completed years of formal schooling and further defined as equal to 4 years, 5 to 9 years and ≥10 years of schooling. Marital status was dichotomized into married or in a common-law marriage and single, widowed, divorced or separated. Concerning household income, participants’ monthly incomes were reclassified according to four categories, ranging from ≤250€ to >2000€. Professional status was also recoded into two groups: professionally active (full-time employee, part-time employee or freelancer) and non-active (unemployed, day or disability pensioner, pre-retirement, student or domestic).

#### 2.2.2. Lifestyle and Health-Related Data

Regarding lifestyle data, smoking habits were assessed by asking participants whether they were ex-smokers, current smokers or non-smokers. Alcohol intake was recorded as the frequency of consumption within one year and was summarized using the following groups: never or less than once a month, once a month to six times a week and equal or over once a day. Data regarding health, including hypertension, previous diagnosis and use of antihypertensive drugs, were self-reported.

#### 2.2.3. Physical Activity

Physical activity was evaluated using the Short Form of the International Physical Activity Questionnaire (IPAQ), validated for the Portuguese population [26], comprising the performance of activities over the past seven days. Information on frequency (measured in days per week) and duration (time per day) was assessed for a set of activities, including walking, moderate-intensity activities and vigorous-intensity activities. To determine the level of physical activity, the data collected from IPAQ were converted into metabolic equivalent (MET) minutes/week. According to the sum of walking, moderate and vigorous MET min per week scores, participants’ level of physical activity was classified as ‘high’ if ≥3000 MET minutes/week; ‘moderate’ if ≥600 MET min/week or ‘low’ if <600 MET min/week [27].

#### 2.2.4. Anthropometric Measurements

Anthropometric measurements were collected in accordance with standard procedures [28]. Height was measured using a stadiometer (Seca 2013), and weight was quantified with TANITTA TBF-300; these measures were used to calculate the participants’ body mass index (BMI): normal (<25.0 kg/m^2^), overweight (25.0–29.9 kg/m^2^) and obese (≥30.0 kg/m^2^).

#### 2.2.5. Adherence to the Mediterranean Diet

Adherence to the Mediterranean diet was assessed using the Portuguese Version of the Mediterranean Diet Adherence Screener (MEDAS) [29]. It consists of 14 questions covering the frequency of food consumption and the characteristics of eating habits of this dietary pattern. Each question is scored with zero or one point, with one point assigned for responses that align with the principles of the MedDiet. A final score ≥10 indicates a good adherence to the MedDiet.

#### 2.2.6. Urinary Sodium and Potassium

A 24-hour urine sample was collected by each participant, who received detailed oral and written instructions on the collection and storage procedures from study interviewers during the evaluation. A container was supplied for the urine collection (3 L), which needed to be kept in the refrigerator until it was delivered. Participants were asked to exclude the first-morning void while collecting all urine over the next 24 hours, including the first void on the following morning. Participants were responsible for delivering their urine samples to the Pedro Hispano Hospital, where the laboratory analyses were performed, namely: urine volume (mL), urinary creatinine (mg/dL) and urine sodium and potassium (mEq/24 h) excretion.

For participants aged ≥65 years old, a 24-hour urine sample was considered complete if the creatinine level was >0.4 g/day for women and >0.6 g/day for men [30] or if the volume collected was >500 mL [31]. In the case of participants <65 years old, the validity of the 24-hour urine sample was evaluated based on the ratio of urinary creatinine to body weight. Samples from females were included when the creatinine (mg/day/kg) ranged from ≥10.8 and ≤25.2, while for males, criteria were met when creatinine (mg/day/kg) was ≥14.4 and ≤33.6 [32].

Sodium and potassium urinary excretion were converted from milliequivalents (mEq) to milligrams (mg) by multiplying it by its atomic weight of 23 or 39 (mg = mEq × atomic weight), respectively. In addition, to estimate dietary sodium and potassium intake, we assumed that approximately 91% and 77% of the consumed sodium and potassium values, respectively, are excreted via urine [33]. Thus, dietary intake of these nutrients was further calculated by applying the following formula: (urinary excretion × 100)/proportion of the consumed nutrients recovered in urine. The molar 24-hour urine Na/K ratio was calculated by dividing the sodium by the potassium.

The prevalence of sodium and potassium intake inadequacy was calculated based on WHO recommendations [5,6].

### 2.3. Statistical Analysis

Normality was evaluated through visual inspection (histograms, box plots and Normal Q-Q plots). Descriptive statistics, such as mean and standard deviation or median and interquartile range, according to the distribution of the variables, were calculated to describe the participants’ baseline characteristics. Categorical variables were reported as absolute and relative frequencies (n and %).

Participants were compared regarding sociodemographic, lifestyle and health-related characteristics, as well as physical activity, according to adherence to the MedDiet, and sodium and potassium excretion, using the chi-squared test or exact Fisher test, as applicable. A comparison of sodium, potassium and Na/K ratio urinary excretion according to the adherence to the MedDiet was also performed using the Mann–Whitney test.

To quantify the magnitude of the association between sodium, potassium and Na/K molar ratio (dependent variables) and adherence to the MedDiet (independent variable), odds ratios (OR) and respective 95% confidence intervals (95% CI) were calculated using separate models of binary logistic regression, adjusted for sex, age, education and use of antihypertensive drugs. The dependent variables in each logistic regression model (sodium, potassium and molar Na/K ratio) were dichotomized according to the median.

All statistical analyses were conducted using version 29.0. of the Statistical Package for the Social Sciences. Statistical significance of the results was considered when the *p*-value < 0.05.

## 3. Results

### 3.1. Characteristics of Participants

A total of 169 subjects, with a median age of 70 years old (ranging from 36 to 85 years of age), were included in this analysis; 60.9% were female. Regarding the presence of hypertension, 56.2% of the participants reported having a previous diagnosis, and 54.2% stated they were taking antihypertensive drugs.

The participants’ characteristics according to MedDiet adherence are described in Table 1. Within this sample, 18.3% of the participants showed good adherence to MD (n = 31). A higher proportion of participants being married or cohabiting was observed among those with good adherence to the MedDiet (87.1% vs. 12.9%, *p* = 0.047). No statistically significant differences were found for the remaining studied sociodemographic, lifestyle, health-related and physical activity variables.

The prevalence of inadequate sodium intake (≥2000 mg/day) was 88.2% (n = 149), without statistically significant differences according to MedDiet adherence. Regarding potassium, 64.5% (n = 109) of participants had insufficient intake (<3510 mg/day), which was higher among individuals with low adherence to the Mediterranean dietary pattern (54.8% vs. 31.2%, *p* = 0.021).

The characteristics of participants classified into sodium and potassium intake groups are presented in Table 2. Those with an insufficient potassium intake were more likely to be female (*p* = 0.014), less educated (*p* = 0.003) and more sedentary (*p* = 0.034).

### 3.2. Sodium and Potassium Intake Molar Na/K ratio and MedDiet Adherence

The median sodium and potassium intake was 3210 ± 1453 and 3150 ± 1256 mg/day, respectively, as shown in Table S1. Descriptive statistics for sodium and potassium intake, as well as the Na/K ratio, according to adherence to the MedDiet are described in Table 3. Individuals with good adherence to the MedDiet were more likely to have higher sodium (3488 vs. 3033 mg/day, *p* = 0.041) and potassium intake (3566 vs. 3036 mg/day, *p* < 0.001). No statistically significant differences were found for the molar Na/K ratio when comparing individuals adhering to the MedDiet with those who did not.

Table 4 shows the association between adherence to the MedDiet and estimated nutrient intake (sodium, potassium) and Na/K ratio. A good adherence to the MedDiet was associated with higher levels of sodium (OR = 3.11; 95% CI: 1.27–7.65) and potassium intake (OR = 9.74; 95% CI: 3.14–30.26). No significant association was observed between adhering to the MedDiet and Na/K ratio.

## 4. Discussion

In this study, good adherence to the MedDiet was associated with a higher sodium and potassium intake.

The published literature regarding sodium intake and adherence to this dietary pattern has revealed heterogeneous findings. In a previous study [34], also conducted in an older Portuguese sample and using both 24-hour urinary excretion and the MEDAS, excessive sodium excretion (≥2000 mg/day) was associated with good adherence to the MedDiet among male participants. Other studies [35,36,37,38] also suggested that higher adherence to this dietary pattern can result in a greater amount of ingested sodium, although intake was measured using food frequency questionnaires (FFQ), and several MedDiet scores were used among these studies. Another study [15] conducted in adults did not find any association between adherence to the MedDiet and urinary sodium excretion. The latter assessed the adherence to this dietary pattern with the alternative MedDiet score calculated from FFQ. The discrepancies among these findings may suggest that the results depend on the methods used to evaluate adherence to the MedDiet. Although the MEDAS is a validated tool to assess adherence to this dietary pattern [29], we cannot neglect that self-reported data may raise social desirability bias, making it important that future research focus on a deeper understanding of these differences. The heterogeneity of the results observed in the literature can also be explained by other differences in the methodologies, such as the use of various scores or indexes to assess adherence to the MedDiet as well as methodological differences regarding the assessment of sodium. Other possible explanations may include different cut-offs and the diversity of the studied populations, which can have different eating habits based on their specific characteristics and ages. Even within the MedDiet, there can be considerable variability regarding sodium intake due to added salt and the consumption of processed foods, which are difficult to measure through dietary pattern adherence scores, such as the MEDAS, since no question can be used as a proxy for this intake. 

The stronger association between adherence to the Mediterranean pattern and potassium intake compared to sodium intake, despite both being statistically significant, may reflect the concept of the Mediterranean diet itself. This dietary pattern is characterized by a high consumption of fruits, vegetables, legumes and nuts, which naturally contain potassium. Moreover, the consumption of these items is assessed in the MEDAS instrument, unlike sodium sources, which are not queried.

Additionally, in this questionnaire, there is no question on the ingested amount of “minimally processed, seasonal, fresh and local foods”, one of the MedDiet’s hallmarks. Also, in our study, data in Table A1 show that the amounts of olive oil, fresh fruit, wine, pulses and nuts consumed do not meet the MedDiet’s criteria. This may imply that the modern MedDiet, or at least that of the population surveyed, is not a traditional MedDiet and does not meet its original definition.

Our sample is considerably heterogeneous in terms of age and education, primarily due to the established inclusion criteria of the MIND-Matosinhos project. However, most of the individuals included in this analysis were older adults (≥65 years), which accounts for the high proportion of individuals with a low level of education (equal to four years). This is consistent with the social context of Portugal in the decades of 50 and 60, where the mandatory level of education was only extended to six years in 1964, which explains the low number of schooling years in almost 40% of the participants.

The aging process is associated with a variety of physiological, psychological and social changes that can lead to poor eating habits [39]. In our study, excessive sodium intake and insufficient potassium intake were observed. This agrees with the previous literature, including data from Portuguese older adults, which showed that most had inadequate sodium and potassium excretion [40]. In the older population, declining gustatory function is very common and may affect their dietary intake, thereby negatively impacting their health. Changes in taste perception are primarily caused by declining chemosensory perception, increasing difficulties in maintaining oral health and changes in olfactory function. A weaker perception of salty flavors may lead people to season their food with excessive amounts of salt, thus increasing the risk of cardiovascular disease [41]. An adequate awareness about the excessive intake of this nutrient can be particularly important among people with a known high risk of dementia to prevent this condition, namely with vascular cause.

The results of this study strongly suggest that a higher adherence to the MedDiet is associated with a higher potassium intake. This is particularly relevant given that a recent study [15], also using a validated score to evaluate the adherence to the MedDiet and 24-hour urinary collections, found no association between these two parameters. However, another study [42] using the same urinary method suggested that higher adherence to this dietary pattern is associated with greater potassium excretion, aligning with the principles of the MedDiet, a major dietary pattern in terms of health, characterized by a high vegetable and fruit consumption. These discrepancies may also be due to different nutrient cut-offs used in the analysis and the diversity of the studied populations, which highlight the importance of developing more studies on these topics using validated and gold-standard methods.

This study has focused on a relevant topic for public health since the MedDiet’s definition often ignores sodium intake and its acceptable amount. This topic should be included in the recommendations for following a healthy dietary pattern, such as the MedDiet [43], which is particularly pertinent when discussing dementia prevention. Given its potential to prevent dementia, the WHO has recommended the MedDiet to adults with normal cognition and mild cognitive impairment [44] as a way to reduce the risk of its onset. Accumulating evidence suggests a link between lower sodium intake and improved cognitive health [45]. However, the findings are mixed, which may be attributed to the use of a wide variety of methods to evaluate dietary sodium intake and cognitive function as well as the heterogeneity of study populations [45]. Hypertension is widely recognized as a significant risk factor for dementia [46], indicating a pathway between salt consumption and adverse cardiovascular and cerebrovascular outcomes that may ultimately lead to cognitive decline and dementia. Nonetheless, the literature has emphasized that dietary sodium may be an independent risk factor for dementia [47,48]. Indeed, a recent study [47] showed that excessive salt intake, measured by 24-hour urine collections, was significantly associated with a faster progression of cognitive impairment and an increased risk of dementia in older adults, independent of other important risk factors, such as hypertension. Additionally, another study conducted in Japan [49] revealed that a higher dietary intake of potassium, assessed through a semiquantitative FFQ, was associated with a lower risk of dementia among older adults. These findings add important evidence to the necessity of reducing sodium consumption to prevent dementia and emphasize the benefits of consuming potassium-rich foods.

Some limitations of the present study must be acknowledged. Data regarding participants’ energy intake were unavailable, and the quantity of ingested food can influence the amounts of excreted sodium and potassium. In fact, a higher quantity of food ingested could explain a greater excretion of these nutrients. It has been shown [50,51] that when nutrients are adjusted for energy intake, different results can be observed, namely an increase in the estimated potassium intake and a decrease in sodium intake. Another limitation was the inclusion of self-reported data, which, as mentioned, may be affected by social desirability bias. It is also important to refer to the limited sample size and the small proportion of participants with good adherence to the MedDiet, which may limit the internal validity of our study.

Nevertheless, we found statistically significant associations between a good adherence to the MedDiet and higher sodium and potassium intake, which highlight the importance of this study. Our results provide crucial information for public health interventions since evidence regarding this relation is still scarce.

Despite the limitations noted, important strengths should be highlighted, such as the use of the gold-standard method for estimating sodium intake, the 24-hour urinary excretion and the use of a validated questionnaire to evaluate adherence to the MedDiet, the MEDAS questionnaire. Additionally, this study was based on a community sample at a higher risk of dementia, for which sodium intake assumes particular relevance given the link between higher blood pressure and dementia [11,12]. However, our findings should not be directly generalized to other adults, namely those who are not at a high risk of dementia. We must acknowledge that people at a higher risk of dementia due to the concomitant presence of several risk factors for this condition may have their eating habits altered more recently or may be more susceptible to bias in self-reported data because of the awareness of their own risk.

Our findings suggest the need to create public health measures to reduce excessive sodium intake, especially among more vulnerable people. Providing nutritional education and training in cooking skills through strategies such as culinary medicine [52] may contribute to improve this outcome. Also, encouraging the population to follow healthy eating patterns, such as the MedDiet, is important to achieve adequate intake levels of relevant nutrients to ensure a good health status, such as potassium.

## 5. Conclusions

The present study has shown that a good adherence to the MedDiet is associated with greater levels of both sodium and potassium intake. However, merely following the MedDiet, typically recognized as a healthy dietary pattern, may not suffice to meet the recommended intake levels of certain nutrients, including sodium. Therefore, public health interventions should promote population awareness regarding salt intake, especially among older people, namely by improving consumer empowerment to an adequate sodium intake. Moreover, promoting healthy eating habits is essential to ensure a sufficient intake of vital nutrients, such as potassium.

## Figures and Tables

**Table 1:** Sociodemographic, lifestyle, health-related characteristics and physical activity of 169 Portuguese adults at a high risk of dementia from the MIND-Matosinhos study (2020–2023), according to Mediterranean Diet adherence.

Sociodemographic, Lifestyle and Health-Related Characteristics and Physical Activity		All Participants n (%)	Good Adherence to the MedDiet (≥10 Points) n (%)	Low Adherence to the MedDiet (<10 Points) n (%)	*p*
		169 (100)	31 (18.3)	138 (81.7)	
Sex	Female	103 (60.9)	18 (58.1)	85 (61.6)	0.839
	Male	66 (39.1)	13 (41.9)	53 (38.4)	
Age (years)	<65	39 (23.1)	5 (16.1)	34 (24.6)	0.594
	65–74	84 (49.7)	17 (54.8)	67 (48.6)	
	≥75	46 (27.2)	9 (29.0)	37 (26.8)	
Education (years)	4	64 (37.9)	11 (35.5)	53 (38.4)	0.710
	5–9	55 (32.5)	12 (38.7)	43 (31.2)	
	≥10	50 (29.6)	8 (25.8)	42 (30.4)	
Marital status ^#^	Married or cohabiting	122 (72.6)	27 (87.1)	95 (69.3)	0.047
	Single, divorced, widowed or separated	46 (27.4)	4 (12.9)	42 (30.7)	
Household income ^#^	250–1000 €	41 (26.1)	4 (12.9)	37 (29.4)	0.240
	1001–1500 €	50 (29.6)	11 (35.5)	39 (31.0)	
	1501–2000 €	30 (19.1)	6 (19.4)	24 (19.0)	
	>2000 €	36 (22.9)	10 (32.3)	26 (20.6)	
Professional status ^#^	Professionally active	20 (12.0)	3 (10.0)	17 (12.4)	1.000
	Professionally non-active	147 (88.0)	27 (90.0)	120 (87.6)	
Physical activity level	High	9 (5.3)	0 (0.0)	9 (6.5)	0.169
	Moderate	83 (49.1)	19 (61.3)	64 (46.4)	
	Low	77 (45.6)	12 (38.7)	65 (47.1)	
Smoking status	Non-smoker	86 (50.9)	20 (64.5)	66 (47.8)	0.238
	Current smoker	13 (7.7)	2 (6.5)	11 (8.0)	
	Ex-smoker	70 (41.4)	9 (29.0)	61 (44.2)	
Drinking frequency (for any alcoholic beverage)	Never or <1/month	32 (18.9)	6 (19.4)	26 (18.8)	0.879
	≥1/month to <1/day	61 (36.1)	10 (32.3)	51 (37.0)	
	≥1/day	76 (45.0)	15 (48.4)	61 (44.2)	
Hypertension diagnosis	No	74 (43.8)	16 (51.6)	58 (42.0)	0.423
	Yes	91 (56.2)	15 (48.4)	80 (58.0)	
Use of antihypertensive drugs	No	77 (45.8)	17 (54.8)	60 (43.8)	0.320
	Yes	91 (54.2)	14 (45.2)	77 (56.2)	
BMI (kg/m^2^)	Normal (<25.0)	42 (24.9)	6 (19.4)	36 (26.1)	0.223
	Overweight (25.0–29.9)	80 (47.3)	19 (61.3)	61 (44.2)	
	Obesity (≥30.0)	47 (27.8)	6 (19.4)	41 (29.7)	
Sodium intake (mg/day)	Excessive (≥2000)	149 (88.2)	29 (93.5)	120 (87.0)	0.537
	Adequate (<2000)	20 (11.8)	2 (6.5)	18 (13.0)	
Potassium Intake (mg/day)	Insufficient (<3510)	109 (64.5)	14 (45.2)	95 (68.8)	0.021
	Sufficient (≥3510)	60 (35.5)	17 (54.8)	43 (31.2)	

BMI: Body Mass Index; MedDiet: Mediterranean Diet. ^#^ the sample does not include 169 individuals in this variable due to missing values.

**Table 2:** Sociodemographic, lifestyle and health-related characteristics and physical activity of 169 Portuguese adults at a high risk of dementia from the MIND-Matosinhos study (2020–2023), according to sodium and potassium intake.

Sociodemographic, Lifestyle and Health-Related Characteristics and Physical Activity		Sodium Intake			Potassium Intake		
		Adequate (<2000 mg/day) n (%)	Excessive (≥2000 mg/day) n (%)	*p*	Sufficient (≥3510 mg/day) n (%)	Insufficient (<3510 mg/day) n (%)	*p*
Sex	Female	15 (75.0)	88 (59.1)	0.224	29 (48.3)	74 (67.9)	0.014
	Male	5 (25.0)	61 (40.9)		31 (51.7)	35 (32.1)	
Age (years)	<65	1 (5.0)	38 (25.5)	0.095	15 (25.0)	24 (22.0)	0.692
	65–74	11 (55.0)	73 (49.0)		31 (51.7)	53 (48.6)	
	≥75	8 (40.0)	38 (25.5)		14 (23.3)	32 (29.4)	
Education (years)	4	6 (30.0)	58 (38.9)	0.097	20 (33.3)	44 (40.4)	0.003
	5–9	4 (20.0)	51 (34.2)		29 (48.3)	26 (23.9)	
	≥10	10 (50.0)	40 (26.8)		11 (18.3)	39 (35.8)	
Marital status ^#^	Married or cohabiting	15 (75.0)	107 (72.3)	1.000	47 (78.3)	75 (69.4)	0.279
	Single, divorced, widowed or separated	5 (25.0)	41 (27.7)		13 (21.7)	33 (30.6)	
Household income ^#^	250–1000 €	3 (20.0)	38 (26.8)	0.296	18 (31.6)	23 (23.0)	0.647
	1001–1500 €	5 (33.3)	45 (31.7)		17 (29.8)	33 (33.0)	
	1501–2000 €	1 (6.7)	29 (20.4)		11 (19.3)	19 (19.0)	
	≥2000 €	6 (40.0)	30 (21.1)		11 (19.3)	25 (25.0)	
Professional status ^#^	Professionally active	0 (0.0)	20 (13.6)	0.135	8 (13.3)	12 (11.2)	0.804
	Professionally non-active	20 (100.0)	127 (86.4)		52 (86.7)	95 (88.8)	
Physical activity level	High	2 (10.0)	7 (4.7)	0.439	6 (10.0)	3 (2.8)	0.034
	Moderate	11 (55.0)	72 (48.3)		33 (55.0)	50 (45.9)	
	Low	7 (35.0)	70 (47.0)		21 (35.0)	56 (51.4)	
Smoking status	Non-smoker	7 (35.0)	79 (53.0)	0.212	29 (48.3)	57 (52.3)	0.446
	Current smoker	3 (15.0)	10 (6.7)		3 (5.0)	10 (9.2)	
	Ex-smoker	10 (50.0)	60 (40.3)		28 (46.7)	42 (38.5)	
Drinking frequency (for any alcoholic beverage)	Never or <1/month	4 (20.0)	28 (18.8)	0.829	8 (13.3)	24 (22.0)	0.310
	≥1/month to <1/day	6 (30.0)	55 (36.9)		25 (41.7)	36 (33.0)	
	≥1/day	10 (50.0)	66 (44.3)		27 (45.7)	49 (45.0)	
Hypertension diagnosis	No	7 (35.0)	67 (45.0)	0.476	30 (50.0)	44 (40.4)	0.258
	Yes	13 (65.0)	82 (55.0)		30 (50.0)	65 (59.6)	
BMI (kg/m^2^)	Normal (<25.0)	7 (35.0)	35 (23.5)	0.532	13 (21.7)	29 (26.6)	0.187
	Overweight (25.0–29.9)	8 (40.0)	72 (43.8)		34 (56.7)	46 (42.2)	
	Obesity (≥30.0)	5 (25.0)	42 (28.2)		13 (21.7)	34 (31.2)	

BMI: Body Mass Index. ^#^ the sample does not include 169 individuals in this variable due to missing values.

**Table 3:** Descriptive statistics of sodium and potassium intake and molar Na/K ratio according to adherence to the MedDiet in 169 Portuguese adults at a high risk of dementia from the MIND-Matosinhos study (2020–2023).

Nutrients	Low Adherence to the MedDiet (<10 Points) Median ± IQR	Good Adherence to the MedDiet (≥10 Points) Median ± IQR	*p*
Sodium (mg/day)	3033 ± 1548	3488 ± 1011	0.041
Potassium (mg/day)	3036 ± 1350	3566 ± 1094	<0.001
Na/K ratio	1.70 ± 0.74	1.58 ± 0.82	0.081

MedDiet: Mediterranean Diet; Na/K ratio: Sodium and Potassium Ratio; IQR: Interquartile Range.

**Table 4:** Association between sodium and potassium intake and molar Na/K ratio and adherence to the Mediterranean diet in 169 Portuguese adults at a high risk of dementia from the MIND-Matosinhos study (2020–2023).

		Crude OR (95% CI)	Adjusted OR * (95% CI)
Sodium	Below the median (<3210 mg/day)	1	1
	Above the median (≥3210 mg/day)	3.00 (1.29–6.98)	3.11 (1.27–7.65)
Potassium	Below the median (<3150 mg/day)	1	1
	Above the median (≥3150 mg/day)	9.31 (3.10–28.06)	9.74 (3.14–30.26)
Na/K ratio	Below the median (<1.69)	1	1
	Above the median (≥1.69)	0.63 (0.28–1.37)	0.59 (0.26–1.30)

* Adjusted for sex, age (continuous variable), education (continuous variable) and taking antihypertensive drugs. Na/K ratio: Sodium and Potassium Ratio; OR: Odds Ratio; 95% CI: 95% Confidence Interval.

## Data Availability

The raw data supporting the conclusions of this article will be made available by the authors upon request.

## Supplementary Materials

The following supporting information can be downloaded at: https://www.mdpi.com/article/10.3390/nu16101419/s1, Table S1. Descriptive statistics of estimated nutrient intake (sodium and potassium) and molar Na/K ratio in 169 Portuguese adults at a high risk for dementia from the MIND-Matosinhos study (2020–2023). Table S2. Descriptive statistics of MEDAS score in 169 Portuguese adults at a high risk for dementia from the MIND-Matosinhos study (2020–2023).

## Appendix A

**Table A1:** MEDAS score results of 169 Portuguese adults at a high risk for dementia from the MIND-Matosinhos study (2020–2023).

MEDAS Score			
Question	Criteria for 1 Point	Yes n (%)	No n (%)
Do you use olive oil as your main culinary fat?	Yes	161 (95.3)	8 (4.7)
How many tablespoons of olive oil do you consume per day?	≥4 tablespoons	14 (8.3)	155 (91.7)
How many vegetables servings do you consume per day?	≥2 servings	86 (50.9)	83 (49.1)
How many servings of fresh fruit do you consume per day?	≥3 servings	70 (41.4)	99 (58.6)
How many servings of red meat, or red meat products do you consume per week?	≤6 servings	165 (97.6)	4 (2.4)
How many servings of butter, margarine, or cream do you consume per day?	<1 serving	99 (58.6)	70 (41.4)
How many sweet/fizzy beverages/sodas do you consume per day?	<1 serving	162 (95.9)	7 (4.1)
How many glasses/cups of wine do you consume per week?	≥7 servings	52 (30.8)	117 (69.2)
How many servings of pulses do you consume per week?	≥3 servings	13 (7.7)	156 (92.3)
How many servings of fish or shellfish do you consume per week?	≥3 servings	103 (60.9)	66 (39.1)
How many times per week do you consume industrial (not homemade) desserts/sweets/pastries?	<3 times	112 (66.3)	57 (33.7)
How many servings of (unsalted) nuts do you consume per week?	≥3 servings	21 (12.4)	148 (87.6)
Do you preferentially consume chicken, turkey or rabbit meat, or a vegetarian protein source, instead of red meat or any derived products?	Yes	137 (81.1)	32 (18.9)
How many times per week do you consume dishes cooked with tomato or tomato sauce, onion and (or) garlic, and olive oil?	≥2 times	141 (83.4)	28 (16.6)

MEDAS: Mediterranean Diet Adherence Screener.
