# Supplementary material for: Adherence to the Mediterranean Diet, Sodium and Potassium Intake in People at a High Risk of Dementia
Source: Nutrients. 2024 May 8;16(10):1419. doi: 10.3390/nu16101419 (PMC11124415; doi:10.3390/nu16101419)
Supplement: Supplementary file 1 [file nutrients-16-01419-s001.zip › nutrients-2957710-supplementary.pdf]

Table S1. Descriptive statistics of estimated nutrient intake (sodium and potassium) and molar Na/K ratio in 169 Portuguese adults at high risk for dementia from the MIND-Matosinhos study (2020–2023).

Sodium Intake (mg/day)			
	**Overall**	**Women**	**Men**
Median ± IQR	3210 ± 1453	2831* ± 1264	3564* ± 1377
Minimum	1289	1289	1340
Maximum	7052	7052	6293
Percentile 25	2502	2325	3109
Percentile 75	3955	3589	4486
**Potassium Intake (mg/day)**			
	**Overall**	**Women**	**Men**
Median ± IQR	3150 ± 1256	2983* ± 1200	3388* ± 1160
Minimum	1114	1114	1378
Maximum	6073	6073	6048
Percentile 25	2634	2396	2955
Percentile 75	3890	3596	4115
**Na/K Ratio**			
	**Overall**	**Women**	**Men**
Median ± IQR	1.69 ± 0.71	1.66 ± 0.80	1.85 ± 0.70
Minimum	0.66	0.72	0.66
Maximum	3.48	3.48	3.02
Percentile 25	1.40	1.29	1.47
Percentile 75	2.11	2.08	2.17

IQR: Interquartile range. * The medians were statistically different by sex.

Table S2. Descriptive statistics of MEDAS score in 169 Portuguese adults at high-risk for dementia from the MIND-Matosinhos study (2020–2023).

Adherence to the Mediterranean Diet (MEDAS Score)			
	**Overall**	**Women**	**Men**
Mean ± SD	7.91 ± 1.78	8.08 ± 1.64	7.64 ± 1.96
Minimum	3	4	3
Maximum	12	12	12
Percentile 25	7	7	6
Percentile 75	9	9	9

SD: Standard deviation.
